# Dizziness and its association with walking speed and falls efficacy among older men and women in an urban population

**DOI:** 10.1007/s40520-019-01303-6

**Published:** 2019-09-05

**Authors:** Ellen Lindell, Lena Kollén, Mia Johansson, Therese Karlsson, Lina Rydén, Anna Zettergren, Kerstin Frändin, Ingmar Skoog, Caterina Finizia

**Affiliations:** 1 Department of Otorhinolaryngology, Head and Neck Surgery, Region Västra Götaland, Sahlgrenska University Hospital, 413 45 Gothenburg, Sweden; 2 Institute of Clinical Sciences, Sahlgrenska Academy, University of Gothenburg, Gothenburg, Sweden; 3 Department of Occupational Therapy and Physiotherapy, Region Västra Götaland, Sahlgrenska University Hospital, Gothenburg, Sweden; 4 Department of Oncology, Region Västra Götaland, Sahlgrenska University Hospital, Gothenburg, Sweden; 5 Neuropsychiatric Epidemiology, Institute of Neuroscience and Physiology, Sahlgrenska Academy, Centre for Ageing and Health (AgeCap), University of Gothenburg, Gothenburg, Sweden

**Keywords:** Dizziness, Unsteadiness, Fear of falling, Falls, Walking speed, Medication

## Abstract

**Background:**

Dizziness is common among older people and falling is a feared complication.

**Aim:**

The purpose of this study was to investigate the presence of dizziness and its association with falls, walking speed and fear of falling, including sex differences, among 79-year-olds. Secondary purposes were to describe the relationship between dizziness and falls to number of medications and diseases.

**Method:**

The study consisted of the fifth cohort of Gothenburg’s H70 birth cohort studies. A sample of 662 79-year-olds (404 women, 258 men) were investigated with questions regarding dizziness, previous falls and falls efficacy [estimated according to the falls efficacy scale Swedish version (FES (S))]. Functional tests included self-selected and maximal walking speed over 20 m.

**Results:**

Dizziness was reported among 51% of the women and by 58% of the men (*p* = 0.12). Approximately, 40% had fallen during the past 12 months (41% women, 38% of the men, *p* = 0.48). Dizziness was related to a higher risk of falls among women (OR 2.63 (95% CI 1.67−4.14, *p* < 0.0001), but not among men (OR 1.07, 95% CI 0.63−1.82, *p* = 0.8). Dizzy individuals had lower scores on FES (S) (*p* < 0.01), more medications (*p* < 0.001) and diseases (*p* < 0.001) than those without dizziness. Participants who reported dizziness walked 10% slower than participants without dizziness (*p* < 0.001).

**Conclusion:**

Women with dizziness more often reported falls compared to women without dizziness—a trend that was not seen among men. Persons with dizziness walked slower. Many medications increased risk of falling; hence, number of medications alone might help pinpoint risk groups for falling.

## Introduction

Dizziness and imbalance are common complaints among older people and increase with advancing age [1]. Dizziness is often reported to be more common among women than among men with approximately 40% of women and 30% of men at age 75 suffering from dizziness or unsteadiness of gait [2, 3]. In fact, as many as 45% of older people living at home report having dizziness [4].

Dizziness among older people can have several etiological factors [5] and is sometimes viewed as a geriatric syndrome: a condition caused by degeneration of multiple functions (vestibular, proprioceptal, visual), also referred to as age- dependent dizziness [6, 7]. However, dizziness might not strictly be age- dependent but rather considered a complex geriatric condition, also associated with depressive symptoms and fatigue [4]. Dizziness and impaired balance have been shown to be more common among those simultaneously suffering from chronic diseases [8]. Other known risk factors for dizziness include locomotor disorders, knee or hip problems, impaired vision, number of medications, benign paroxysmal positional vertigo (BPPV) or vestibular hypofunction [6, 8–10]. Dizzy older people tend to be less active than the non-dizzy [8, 11, 12], potentially due to dizziness and poor balance increasing risk of falling [13]. In Sweden, over 1700 older adults die each year due to fall-related accidents, where stumbling or hitting an obstacle is among the most common causes of falling, followed by dizziness and imbalance [13, 14]. Falls and fall-related injuries cost large amounts annually—a figure that in 2012 reached 25 billion Swedish crowns (SEK) [14]. Women tend to fall and seek medical care due to falls more often than men, but fall-related mortality is higher among men [14]. Reasons for this gender difference are not known nor why women, at least in young ages, more often report dizziness compared to men.

Being dizzy can affect walking speed, which is a potential tool when evaluating health, fitness and mobility. Normal function of gait gives a valuable illustration of multi-systemic function and general wellbeing [15, 16], and low walking speed may predict morbidity and mortality [15–18].

Fear of falling and low fall-related self-efficacy have been shown to influence quality of life in a negative way [19] and to be associated with activity restriction [12], reduced social activity and a high occurrence of depressive disorders, especially among women [20–22]. Fear of falling has also been associated with frailty among older adults [22]; however, the association between fear of falling and walking speed is not well documented, nor is walking speed in relation to dizziness and falls.

Many medications can cause dizziness and exposure to polypharmacy has been associated with dizziness [3] as well as risk of falling [3, 23]. The use of medications has increased remarkably during the past 20 years, where the number of medications taken has increased 100% among older adults [24]. There are some gender differences in drug prescription where women tend to use more medications and also being prescribed inappropriate drugs-like medications with anticholinergic drugs and benzodiazepines with can cause dizziness among older adults [25]. Increased prevalence of medications use also enhances the risks of polypharmacy, side effects and inter-drug interactions.

As the association between dizziness, walking speed and falls is not well documented in larger cohorts of older adults, this study aims to investigate the presence of dizziness, including sex differences, and its association with falls, walking speed and fear of falling among 79-year-olds. Additional aims include analyzing the relationship between dizziness, falls to number of medications and diseases.

## Methods

The study comprised the fifth cohort in the Gothenburg H70 birth cohort studies, Sweden and was conducted in 2009. The study participants were selected according to specific birth dates from an urban population born in 1930. Information regarding date of birth and address was obtained from the Swedish national registration register covering all persons registered in Sweden. Invitation for the study was first sent by letter including consent form and the participants were then contacted by telephone. Participants were considered eligible for the study regardless of the place of living (privet household, sheltered living, etc.) A total of 1063 (659 women, 404 men), all aged 79, were invited and 662 (404 women and 258 men, response rate 62%) agreed to participate in the multidisciplinary study. Reasons for not choosing to participate could not be registered due to lack of consent. Measurements were performed by trained nurses and physiotherapists at the neuropsychiatric outpatient clinic at the Sahlgrenska University Hospital. The design, procedures and methods for data collection have been reported elsewhere [26].

### Study-specific questions

The participants answered the question regarding dizziness: “Do you have any problems with dizziness, unsteadiness or impaired balance?” (yes/no) which divided the participants into groups of having dizziness/imbalance (answering “yes”) and no dizziness imbalance (answering “no”). Specific questions about dizziness were then posed only to the participants reporting dizziness, i.e., about occurrence and duration of dizziness, as well as dizziness according to different positionings/movements. The response alternatives were yes/no. The questions concerning falls were “Have you fallen during the last year? (yes/no)”, “How many times have you fallen?” and “If you have fallen, did you get any injury? (no/fracture/soft tissue damage/other)”.

### Walking speed

Average walking speed among 70-year-olds is 1.10−1.25 m/s and walking speed < 1 m/s is considered a risk of poorer health [27, 28]. Walking speed is a highly valid and reliable test [16]. Walking speed was tested over a distance of 20 m. The time consumed for the distance was measured twice: at self-selected/normal walking and when walking as fast as possible (maximal walking speed).

### Falls efficacy

Fear of falling was evaluated using The falls efficacy scale Swedish version (FES (S)). The falls efficacy scale, originally developed by Tinetti et al. [29], measures self-perceived confidence in task performance in common activities of daily living without falling. The Swedish version, (FES (S)) [30], is a modification of the original FES which includes 13 items. The respondents rated their confidence on a visual 0−10-point scale (0 = not confident at all and 10 = completely confident). The scale includes three parts: six items measuring personal activities of daily living (ADLs), one question regarding walking up and down stairs (question number 7) and six items measuring instrumental activities of daily life (iADLs). A higher score (of a maximum of 130 points) indicates better confidence/self-efficacy in performing the different activities without falling.

### Comorbidity and intake of medication

Regarding diseases, the participants were systematically asked about current conditions and diseases; “Have you been told by a medical doctor or nurse that you have or have had, any of the following diseases or disorders?” and were asked according to a list of conditions including diabetes, high blood pressure, chronic bronchitis, asthma, angina pectoris, cardiac infarction, intermittent claudication, heart failure, sciatic/backpain, stroke, vision and hearing impairment, joint problems. Additionally, participants were asked to list their medications.

### Statistical analyses

The software used for the statistical analyses was a statistical program package developed at the Department of Geriatrics at Gothenburg University (GIDSS for Windows). Differences in dichotomized variables were analyzed with logistic regression or Fischer’s exact test (difference of occurrence of dizziness and falls for men and women). Significance was always reported for two-tailed tests, and the significance level used was 5%. Means were compared between groups using *t* test. Linear regression was used for analyzing association between the number of medication and walking speed.

## Results

In this cohort comprising 662 (404 women, 258 men) mainly home dwelling 79-year-olds, dizziness and/or impaired balance was reported by more than half of the individuals with no significant sex differences (51% women, 58% men, *p* = 0.12). In the majority of the participants (82%), symptoms of dizziness had been present for more than 6 months. Approximately, one-third of the dizzy participants had daily problems with dizziness and more than one quarter (29% of the women and 26% of the men) reported that dizziness had an impact on their daily activities. Dizziness lasting for only seconds was the most commonly reported duration of dizziness and was mainly triggered by rising up from supine position followed by positional changes (Table 1).

**Table 1:** Characteristics of the study 79-year-old population answering questions regarding dizziness

	Female		Male	
	Yes (*n* %)	Total*	Yes (*n* %)	Total^a^
Do you have any problems with dizziness, unsteadiness or impaired balance?	184 (51)	359	141 (58)	243
Participants reporting dizziness		124		82
Duration of dizziness				
Seconds	88 (71)		46 (56)	
Minutes	19 (15)		14 (17)	
Hours	17 (14)		22 (27)	
Problems with dizziness—since how long?		117		77
< 1 month	2 (2)		2 (3)	
2−6 months	22 (19)		9 (12)	
6 months−2 years	34 (29)		26 (33)	
> 2 years	59 (50)		40 (52)	
Dizziness being and obstacle in activities	42 (29)	143	23 (26)	89
Dizziness reported when				
When rising from supine to sitting position	64 (54)	119	43 (54)	80
When sitting	6 (6)	100	5 (7)	71
When lying on the side	2 (2)	98	8 (12)	69
When turning the head	22 (22)	99	18 (25)	73
When tilting the head backwards	22 (22)	102	14 (20)	70
When anxiety	11 (11)	103	10 (14)	69
By positional changes	37 (34)	110	32 (44)	73
By physical exercise	21 (21)	100	15 (21)	73

The question “Do you have any problem with dizziness, unsteadiness or impaired balance” was used to categorize participants as having dizziness or not. Only participants answering “yes” to the first questions were asked the following questions about specific symptoms of dizziness

^a^Total number of participants answering the question

### Falls

Among the 79-year-olds in this study, 40% (247/617) reported at least one fall during the previous year, where 154 (41%) women and 93 (38%) men had fallen (*p* = 0.48). Among those who had experienced three falls or more (*n* = 37), over 80% suffered from dizziness/imbalance. Prior falls were more frequent among women with dizziness/imbalance: odds ratio 2.63 (95% CI 1.67−4.14, *p* < 0.0001), a trend not seen among men; odds ratio 1.07 (95% CI 0.63−1.82, *p* = 0.8). Injuries due to falls were relatively common: 37% of those who had fallen had been injured. In 16% (*n* = 40), the injury was a fracture.

### Walking speed

Both women and men with dizziness/imbalance had a lower walking speed compared to those without dizziness at both self-selected and maximal speed (Table 2). Lower normal walking speed was also seen among men with previous falls (mean 1.11 m/s standard deviation (SD) 0.18 without falls compared to 1.05 m/s SD 0.19 if previously fallen, *p* = 0.05). This observation was not seen among women (mean 1.08 m/s SD 0.18 without falls compared to 1.07 m/s SD 0.20 if previously fallen *p* = 0.63).

**Table 2:** Walking test 20 m at self-selected and maximum speed (m/s) among 79-year-olds, with and without dizziness/imbalance

	Women		Men	
Walking speed mean; m/s (SD)	No dizziness/imbalance (*n* = 104)	Dizziness/imbalance (*n* = 112)	No dizziness/imbalance (*n* = 90)	Dizziness/imbalance (*n* = 86)
Self-selected speed				
No previous fall	1.12 (0.18)	1.01 (0.14)***	1.16 (0.17)	1.07 (0.18)**
*n*	81	54	58	58
Previous fall	1.16 (0.20)	1.03 (0.19)**	1.18 (0.16)	0.98 (0.2)**
*n*	23	58	32	28
Total	1.13 (0.19)	1.02 (0.17)***	1.14 (0.17)	1.04 (0.19)***
Maximal speed				
No previous fall	1.51 (0.26)	1.35 (0.25)***	1.66 (0.27)	1.55 (0.32)*
*n*	81	54	57	58
Previous fall	1.55 (0.28)	1.33 (0.28)**	1.62 (0.205)	1.37 (0.43)**
*n*	23	57	32	27
Total	1.52 (0.27)	1.34 (0.26)***	1.65 (0.27)	1.49 (0.37)**

*P* values based on *t* test comparing dizzy and non-dizzy individuals, *p < 0.05, **p < 0.01, ***p < 0.001

The test was performed over 20 m at self-selected and maximal speed. Mean speed (m/s) for groups with and without dizziness was reported as well as if the participants had fallen the last 12 months

### Falls efficacy

Individuals with dizziness had lower total scores on FES (S) than non-dizzy individuals; mean 112 (SD 26) compared to 120 (SD 20) for women (*p* < 0.01), and 111 (SD 25) compared to 122 (SD 16) for men (*p* < 0.001), indicating less fall-related self-efficacy and higher fear of falling in task performing for dizzy individuals. For women with prior falls, the mean score was 110 (SD 30) compared to 118 (SD 19) among those without prior falls (*p* < 0.01). For men, the corresponding figures were 111 (SD 29) compared to 118 (SD 19), (*p* < 0.01). Both men and women showed relatively high overall self-confidence in performing the different tasks. Nevertheless, the proportion of individuals with maximum scores (128−130p) differed among those with and without dizziness/imbalance (43% compared to 65%, *p* < 0.01). The proportion of individuals with lowest scores (< 112p) also differed among those with and without dizziness/imbalance (34% compared to 14%, *p* < 0.01).

### Comorbidity and intake of medication

Almost half of the individuals used five or more medications daily (49% of women and 43% of men, *p* = 0.23). Number of medications was categorized into four classes 0, 1−4, 5−9 and 10 and more. We found an association between number of medications and dizziness as well as between number of medication and falls (Table 3). The odds ratio of experiencing dizziness associated with each higher class was 1.68 (95% CI 1.26−2.23, *p* < 0.001) for women and 1.49 (95% CI 1.06−2.11, *p* < 0.05) for men. The corresponding figure for the association between medication and falls was 1.57 (95% CI 1.19−12.07, *p* < 0.01) for woman and 1.46 (95% CI 1.03−2.05, *p* < 0.05) for men. Among those taking 10 or more medications, more than half of the persons had fallen and 10% of the women and 28% of the men had fallen three times or more during the past year. There was a linear trend between number of medications and lower walking speed (normal and maximal speed,) among both men and women (*p* < 0.0001).

**Table 3:** Number of medications and previous falls

	Women (*n* = 376)			Men (*n* = 246)		
Number of medications	Falls no (%)	Falls yes (%)	*P* value	Falls no (%)	Falls yes (%)	*P* value
0	22 (76)	7 (24)		12 (57)	9 (43)	
1−4	105 (64)	59 (36)		81 (68)	38 (32)	
5−9	79 (53)	70 (47)		49 (57)	37 (43)	
> 9	15 (44)	19 (56)		7 (35)	13 (65)	
Total	221	155	< 0.01	152	97	< 0.05

*P* values based on logistic regression was used; differences between participants fallen and not fallen during the last 12 months and increasing number of medications

More than half of the study participants (59%) suffered from high blood pressure, which was the most common condition and more common among men with dizziness than among men without dizziness. Joint problems and vision impairment were more commonly found in both genders among persons reporting dizziness compared to those without (Table 4). Heart failure and self-reported hearing impairment were more common among women with dizziness than women without dizziness. No gender differences were seen regarding number of diseases, mean 3.6 (SD 2.25) for men and 3.5 (SD 2.25) for women, *p* = 0.5. People with dizziness suffered from a higher number of diseases; mean 4.0 (SD 2.38) compared to 3.0 (SD 2.0) for those without dizziness, *p* < 0.001. The odds ratio of having dizziness was 1.30 (95% CI 1.17−1.43, *p* < 0.001) for every added disease. Corresponding figure for having experienced falls was 1.15 (95% CI 1.04−1.28, p < 0.01) for every added disease.

**Table 4:** Frequency of conditions and diseases among women and men with and without dizziness

	Women			Men		
	Non-dizzy *n* (%)	Dizzy *n* (%)	Odds ratio (CI)	Non-dizzy *n* (%)	Dizzy *n* (%)	Odds ratio (CI)
Diabetes	16 (10)	27 (13)	1.33 (0.69−2.56)	19 (16)	18 (15)	0.93 (0.46−1.87)
Hypertension	95 (59)	119 (57)	0.93 (0.62−1.42)	64 (53)	82* (67)	1.75 (1.04−2.94)
Lung disease	21 (13)	28 (13)	1.03 (0.56−1.88)	13 (11)	18 (15)	1.42 (0.66−3.05)
Angina pectoris	9 (6)	21 (10)	1.89 (0.84−4.24)	9 (8)	13 (11)	1.45 (0.60−3.55)
Cardiac infarction	10 (6)	13 (6)	0.99 (0.42−2.32)	14 (12)	23 (19)	1.76 (0.86−3.60)
Claudicatio intermittens	1 (0,6)	9 (4)	7.03 (0.88−56.1)	9 (8)	7 (6)	0.74 (0.27−2.05)
Heart failure	16 (10)	47** (22)	2.61 (1.42−4.81)	23 (19)	30 (25)	1.37 (0.74−2.54)
Stroke/TIA	21 (13)	36 (17)	1.39 (0.78−2.49)	32 (28)	29 (24)	0.85 (0.47−1.51)
Vision impairment	41 (33)	70* (49)	1.92 (1.17−3.16)	29 (31)	42* (47)	1.93 (1.06−3.53)
Hearing impairment	43 (35)	73** (51)	1.92 (1.17−3.15)	41 (44)	44 (48)	1.19 (0.66−2.12)
Joint problems	44 (35)	89** (62)	3.00 (1.18−4.94)	22 (24)	37* (41)	2.25 (1.19−4.26)
Back pain	57 (47)	89* (62)	1.85 (1.13−3.03)	29 (31)	40 (45)	1.80 (0.98−3.30)

*P* values based on logistic regression comparing dizzy and non-dizzy individuals, *p < 0.05, **p < 0.01

*CI* confidence interval

## Discussion

Over 50% of 79-year-old men and women reported dizziness and/or impaired balance, with no gender differences. Persons with dizziness walked slower, and women tended to report more falls than men.

At age 75, when the cohort was previously investigated, only 30% of the men and 40% of the women reported problems with dizziness [12], where the increase especially among the men at the age of 79 is a bit hard to explain, but could be due to the higher age of our participants. An explanation why dizziness has increased more among the men could be that the men suffered from a higher burden of disease compared to women in this age. However, the number of diagnosed diseases or medications taken was not higher among the men. Men are generally considered healthier and perform better in physical tests, but still mortality rate is higher in males compared to females especially in high ages, and life expectancy is higher among women. Oksuzyan et al. [31] proposed the theory that women go from “healthy” to “unhealthy” in younger ages than men, but tend to live longer as “unhealthy”. Possibly, this shift might be in the age around 79, targeting the life expectancy of 80 among Swedish men [32].

However, comorbidity and disease burden may be more complex than just diagnoses, with great variations and severity of the conditions creating an impact on the individual not quantified in this study. Tamber et al. [3] has shown that associations between number of medications, diseases and dizziness are at the same level among young and elderly persons in multivariate analyses, when controlling for dizziness [14]. This supports the theory of equal comorbidity between men and woman in this study. Dizziness is considered more common among women and female predominance is reported in almost every survey investigating dizziness. This study reflects the frequency of dizziness/unsteadiness in a general older adult population and not merely patients seeking medical care due to symptoms of dizziness. Physicians meeting older people should be aware of the fact that dizziness is equally common among both genders in higher ages and ask for symptoms of dizziness, even if this is not the main reason for the consultation, as dizziness and impaired balance may have impact on overall wellbeing and quality of life.

From a clinical standpoint, the most commonly reported duration of dizziness was seconds and the most common triggers of dizziness symptoms (after rising from supine to sitting) were positional changes and head movements like turning or tilting the head backwards (Table 1). These symptoms are often prominent in benign paroxysmal positional vertigo (BPPV), which is common among older people and may create a sense of unsteadiness [10]. Since BPPV is a cause of dizziness that can be cured if treated, physicians should actively search for BPPV among older people reporting dizziness by positional changes [33].

Approximately, 40% of the participants had experienced at least one fall during the past year which is in line with previous reports regarding falls [34]. Women experiencing dizziness reported more falls compared to non-dizzy women, but this trend was not observed among men. One possible explanation may be the reported comorbidity, where dizzy females to a greater extent, when compared to non-dizzy females, reported problems with locomotion (i.e., joint problems) proprioceptal and visual impairment—two feedback systems important for maintaining and controlling balance (Table 4). This reduction in locomotion and vision may lead to women being more prone to falling. These comorbidities were not present to the same extent among men, possibly explaining the discrepancy observed in terms of fall tendency.

A low walking speed can be a sign of morbidity and poor physical performance [15] and dizzy participants showed a reduction of walking speed by more than 10%. There was a clear relation between a low walking speed and an increasing number of medications, possibly due to multiple comorbidities that physically limited walking. However, the lower walking speed in persons with dizziness/imbalance could also be caused by a voluntary restriction of speed, stemming from fear of movement to reduce the risk of impaired balance function and falls [12]. Nevertheless, experiencing a previous fall was only moderately associated with decreased walking speed among men. A single fall, if for example induced by slipping or stumbling, may possibly be externalized by the patient, deemed out of their control and independent from their health status, thereby not associating it with a fear of risking repetitive falls.

Both men and women with dizziness had higher fear of falling measured with FES (S). Many of the subscales reached ceiling effects, thereby making it difficult to capture true variance to monitor fall-related risks since the balance confidence overall is high even in this high age group. Instability of gait, impaired fall-related fall efficacy and fear of falling is still not well understood. Herman et al. argue that gait variability as a measure of instability is connected with fear of falling rather than muscle strength and previous falls [35]. Even if lower fall-related efficacy indicating fear of falling was found among persons with dizziness/imbalance and those with previous falls, it is doubtful how valuable the (FES (S)) is as a tool to measure balance confidence or to capture individuals with risk for falls among older adults in general.

Number of medications as a single measure has prior been shown to predict mortality and unplanned hospitalization, especially among men [36, 37]. We found that dizziness as well as falls was associated with increasing number of medications and conditions, which might support the theory that dizziness, as a symptom, is a part of comorbidity and fragility but also that number of medications alone is associated with falls. Polypharmacy itself also may enhance the risk for adverse drug interaction, potentially causing dizziness and fall tendency which is why the reduction of medication is always desirable. The number of medications alone might, therefore, help pinpoint risk groups needing investigation for physical functioning as well as balance enhancing treatment for fall avoidance.

## Limitations

The dual nature of asking about both dizziness and imbalance in the same question poses some limitations. Due to the original design of the longitudinally cross-sectional study, opportunities to change questions does not exist but should be kept in mind for future studies.

More participants completed the questions than those who underwent the walking tests, and not all filled out the Falls Efficacy Scale (S) nor all the questions regarding dizziness. Concerning questions about falls, it may be difficult to recall over a period of one year, thus leading to recall bias. The study has a cross-sectional design making direction of causality difficult. Finally, more women than men were invited in this population-based sample and, therefore, more women than men participated in the survey, which might affect the study’s external validity and reduce the overall generalizability. The selection for participating in the study was based on specific birth dates from an urban population.

## Conclusion

Dizziness at age 79 was reported among more than half of the participants with no gender differences. Women with dizziness more often reported falls compared to women without dizziness—a trend that was not seen among men. Persons with dizziness/imbalance walked slower than those without. Dizziness was associated with increasing number of medications and increased comorbidity. Many medications increased the risk of falling as well as being associated with decreased walking speed; hence, number of medications alone might help pinpoint risk groups for falling.
